# Image guided sclerotherapy for the treatment of venous malformations

**DOI:** 10.1186/s42155-018-0009-1

**Published:** 2018-06-28

**Authors:** Johnathon Gorman, Steven J. Zbarsky, Rebecca J. M. Courtemanche, Jugpal S. Arneja, Manraj K. S. Heran, Douglas J. Courtemanche

**Affiliations:** 1 University of British Columbia, Vancouver, BC V6T 1Z1 Canada; 2 Department of Surgery, University of British Columbia, British Columbia Children’s Hospital, Vancouver, BC V6H 3V4 Canada; 3 Department of Surgery, Division of Plastic Surgery, University of British Columbia, British Columbia Children’s Hospital, K3-131 4480 Oak St., Vancouver, BC V6H 3V4 Canada; 4 Department of Radiology, University of British Columbia, British Columbia Children’s Hospital, Vancouver, BC V6H 3V4 Canada

**Keywords:** Sclerotherapy, Vascular malformations, Venous malformations, Vascular anomalies

## Abstract

**Background:**

Percutaneous sclerotherapy is the preferred method of treatment for VMs (venous malformations). However, treatment protocols vary, and research on the most effective sclerosant is conflicting. Additionally, there is limited knowledge on the effect of sclerosant volume on treatment outcome. This study aims to determine the outcomes and complications of image-guided sclerotherapy for VMs with respect to sclerosant and sclerosant volume. Towards this, a 10-year retrospective chart review was conducted of patients with VMs treated with sclerotherapy at the Vascular Anomalies Clinic at British Columbia Children’s Hospital.

**Results:**

Thirty-four patients with VMs were treated of which 15 had a successful outcome, 13 had a failed outcome, and 6 had additional planned treatments after the study time period. Lesions on the head and neck or classified as type I or II had the highest success rates. Combination therapy with both ethanol and 3% STS (sodium tetradecyl sulphate), and ethanol alone had a higher success rate (64% and 60%) compared to 3% STS (11%). Major complications were most associated with 3% STS (17%) followed by ethanol (9%), and no major complications were found with combination. No relationship between the volume of sclerosant per lesion volume and outcome was found.

**Conclusions:**

Combination and ethanol were the most effective sclerosants in terms of highest success rates and lowest complication rates, and sclerosant volume per lesion volume had no effect on outcomes. Future work should aim at studying larger sample sizes to account for the multiple factors that may influence the choice of sclerosant and treatment outcomes.

## Background

As proposed by Mulliken and Glowacki in 1982, vascular anomalies are categorized based on their endothelial characteristics into tumors and malformations, with malformations sub-classified based on flow and vascular anatomy (Mulliken & Glowacki, 1982). Venous malformations (VMs) are the second most common vascular anomaly and occur with an estimated prevalence of 1% in the general population (Puig et al., 2005; Greene, 2011). These lesions are congenital, grow proportionately with the child, and may present in any anatomical location, with the majority in the craniofacial region (60%) (Ernemann et al., 2010). The presenting signs and symptoms are largely dependent on location and extent of the lesion and most commonly include discoloration, swelling, and pain due to thrombosis (Greene, 2011; Berenguer et al., 1999).

Percutaneous sclerotherapy is the preferred management for VMs, with ethanol and 3% sodium tetradecyl sulfate (STS) used most often. However, reports on the efficacy of sclerosants vary and often conflict (Su et al., 2010; Burrows & Mason, 2004). Ethanol has been associated with the lowest incidence of lesion re-expansion and therefore is suggested as the most effective and first choice for sclerosing agent (Ernemann et al., 2010; Prasetyono & Kreshanti, 2010). However, ethanol has also been cited as being associated with more painful injections and having a higher complication rate than STS, leading many to use STS over ethanol due to reports of it being a safer alternative (Odeyinde et al., 2013; Lee et al., 2003). Most recently, a systematic review highlighted the lack of agreement on which agent is the safest and most effective (Horbach et al., 2016). However, these authors discouraged the use of ethanol for sclerotherapy in the cervicofacial region as this sclerosant had the highest reported complication rates (Horbach et al., 2016). Previous literature also highlights the variability in recommended treatment intervals and dosages. Alakailly et al. reported a dosage methodology of 0.5 mL sclerosant per 2 cm superficial lesion size, whereas others report injecting until resistance is felt (Alakailly et al., 2015; Stuart et al., 2015).

The continued lack of consensus for the optimal sclerotherapy treatment of VMs demonstrates a need for further investigation. We aimed to review and analyse outcomes and complications of image-guided sclerotherapy for the treatment of VMs in patients managed by the Vascular Anomalies Clinic at British Columbia Children’s Hospital (BCCH). Our objectives were to determine which sclerosant was the most effective with respect to outcomes and complications as well as explore the effect of sclerosant volume per lesion volume on outcomes.

## Methods

This study was approved by the University of British Columbia and Children’s and Women’s Hospital Ethics Committee H13–01399. A 10-year retrospective chart review from May 1, 2001 to May 1, 2011 was conducted of VMs managed by the Vascular Anomalies Clinic at BC Children’s Hospital. Data collection included demographic data, VM volume, presenting symptoms, indications for treatments, sclerotherapy treatment details, outcomes, and complications. Patients with no follow-up data were excluded from analysis.

VM volumes were calculated via analysis of three-dimensional imaging. VMs were classified based on interactions with adjacent vasculature, as outlined by Puig et al., (2003) Table 1.

**Table 1:** Classification of VMs according to Puig et al. (2003)

VM type	Lesion characteristics
I	Little to no interaction with adjacent vasculature
II	Drainage of lesion into physiologic venous circulation
III	Drainage of lesion into dysplastic vasculature
IV	Lesion comprised largely of ectatic veins

Sclerotherapy was undertaken with either ethanol alone, 3% STS alone, or targeted treatment with both sclerosants at the same session commonly referred to as combination therapy. All procedures were performed by a single interventional radiologist (M.K.H), using image guided (fluoroscopic) intralesional injection of contrast to confirm location and distribution of each injection followed by non-foamed sclerosant.

In our patients, the sclerosant was chosen to optimize treatment and minimize complications based on clinical experience rather than a strict protocol. The interim outcome following each sclerotherapy session was determined based on the response of the VM measured by its reduction in size and lessening of symptoms. At each follow-up visit, the size change was categorized as complete (> 90% reduction), good (> 50% reduction), moderate (< 50% reduction), no response, or recurrence. Whether the lesion became less symptomatic, asymptomatic, or re-symptomatic was also recorded. It should be noted that interim recurrences may have occurred irrespective of overall outcome.

The overall treatment outcome at 2-years was defined as a success if there was a complete or good response in terms of size change, and the VM was asymptomatic following all treatment sessions. The overall treatment was defined as a failure if the VM had either a moderate or no response in size reduction or was symptomatic following all treatment sessions. When further treatments were scheduled beyond the study time period, the overall outcome was defined as ongoing**.** All of these patients remain in active follow-up in our Vascular Anomalies Clinic.

Sclerotherapy outcomes were reviewed with respect to class and location of VM, number and frequency of treatment sessions, relative volume and type of sclerosant used, as well as recurrence. Sclerosant success rates were calculated by the relative number of times a sclerosant was associated with an overall successful outcome compared to a failed outcome. Complication rates were determined based on the relative number of times an agent was used.

## Results

From the Vascular Anomalies Clinic database 36 patients were identified as having procedures in interventional radiology of which 2 were excluded due to lack of follow-up data. The remaining 34 patients had a total of 82 unique sclerotherapy sessions and were included for analysis. In the study population, there were 25 females and 9 males, with a median age of 15.5 years (SD 14.9, range 2.8–66.1) at the time of their first sclerotherapy treatment. The median follow-up time following a sclerotherapy session was 19.7 weeks and the total average follow-up time was 3.2 years (range 0.1–8.7 years). Ethanol was the most commonly used sclerosant (43/82) followed by 3% STS (24/82), combination of ethanol and 3% STS (14/82), and bleomycin (1/82). Fifteen patients were found to have a successful outcome, 13 had a failed outcome, and 6 required ongoing treatment.

While the majority of patients had no prior therapeutic interventions (30/34) two had previous sclerotherapy, one had surgery, and one had both sclerotherapy and surgery. Indications for treatment were swelling (94%), discoloration (88%), and pain (88%). Also noted were phlebitis (62%), phleboliths (50%), and the presence of a scar (15%). The majority of patients were treated for symptoms (33/34) and one for symptom prevention. Pre-treatment imaging, including MRI, was done in all 34 cases.

**Fig. 1:**
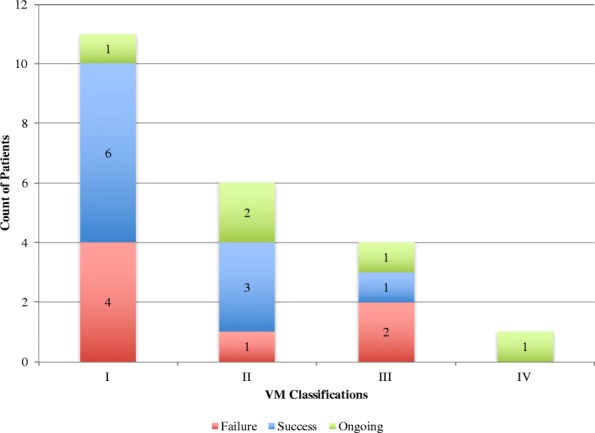
Success and failures of treatment for each class of VM

VMs located in the head and neck (29%) had the highest proportion of successful outcomes (70%), while the two VMs that extended across multiple sites had failed outcomes (Table 2). The types of tissues infiltrated were either deep or superficial subcutaneous, muscle, bone, pleura, parotid gland, and oral mucosa. The most common classes of VMs were Type 1 (32%) and Type II (18%) and were also associated with the most successful outcomes, Fig. 1. The other VMs were classed as Type III (12%) or Type IV (3%) and the remaining 12 VMs (35%) could not be classified, as no digital images were available.

**Table 2:** Distribution of VMs by site and sclerotherapy treatment outcome

Site	Outcome			Total
	Success	Failure	Ongoing	
Head and neck	7	1	2	10
Lower limb	1	5	3	9
Upper limb	4	4	–	8
Trunk	2	1	–	3
Multifocal	1	–	1	2
Extending over multiple sites	–	2	–	2
Total	15	13	6	34

Of the 82 sclerotherapy sessions, 34 were for initial treatment, 36 for planned serial or staged treatment, and 12 for lesion recurrence. Treatments for recurring symptoms following prior treatment were done at an average of 31.9 months (SD 21.8, range 6.6–75.4 months), while treatments that were part of a series occurred at an average interval of 5.5 months (SD 4.8, range 0.16–23.6 months). Serial intervention is planned every 3–6 months depending on the initial response to treatment, but the real interval between treatments is determined by resources, booking, and patient/social factors. Patients with a successful overall outcome had an average of 2.2 sessions (SD 1.4) and those with a failed overall outcome had an average of 2.8 sessions (SD 1.9).

In 16/34 patients, 21 interim recurrences were observed at an average time of 21.5 months (SD = 23.5, range 8.0 weeks – 8.3 years) since the last sclerotherapy session. Recurrences occurred after treatment with ethanol (10), 3% STS (7), and combination (4). Relative to the number of times a sclerosant was used, the recurrence rates were calculated for ethanol (23%), 3% STS (30%), and combination (29%) and there was no statistically significant difference, *p* = 0.843. Of these 16 patients, three had a successful overall outcome, seven had a failed overall outcome and six had ongoing treatments following the study time period.

Of the overall successful outcomes (15 patients), there were a total of 33 treatments with the majority being ethanol (73%), followed by combination (21%), and 3% STS (6%). Of the overall failed outcomes (13 patients), there were a total of 37 treatments, of which the majority were ethanol (43%) and 3% STS (43%), followed by combination (11%), and bleomycin (3%). Combination was found to have the highest success rate with 7/11 (64%) treatments associated with an overall success, followed by ethanol (24/40, 60%), and 3% STS (2/18, 11%). Combination and ethanol were statistically significantly more likely to be associated with a successful outcome than 3% STS, *p* = 0.002.

Of the 13 patients with failed outcomes, seven had no response to treatment, four had an initial good response to treatment with one or more recurrences, and two patients with VMs on the palmar or plantar surface remained symptomatic. The VM on the palmar surface as well as two VMs located on the face with no response were surgically excised.

**Fig. 2:**
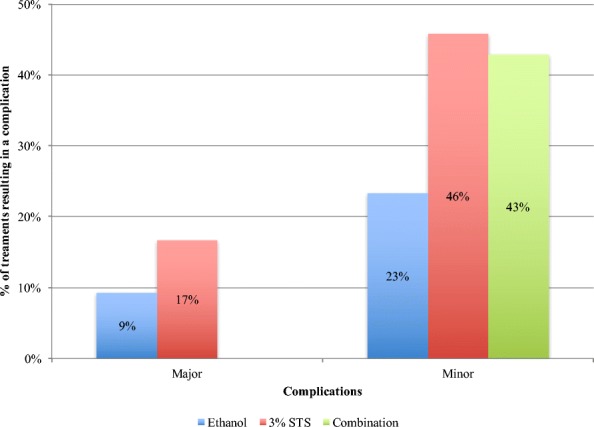
Major and minor complication rate for each sclerosant. Single treatment with bleomycin excluded

There were 14/34 patients who experienced one or more major or minor (self-limiting) complications (41%). In total there were 27 complications observed classified into 8 major and 19 minor (Table 3). Palsy and pain are attributed to perineural injection. Compartment syndrome was due to intramuscular injection of a venous malformation with active localized intravascular coagulopathy. Decreased mobility is attributed to stiffness and scarring in muscle after injection. The major complication rate, was found to be the highest for 3% STS (17%) followed by ethanol (9%). No major complications were associated with combination, Fig. 2.

**Table 3:** Major and minor complications

Complication	Count
Major	
Chronic pain	2
Compartment syndrome	1
Decreased mobility or ROM	4
Nerve palsy (prolonged)	1
Subtotal	8
Minor	
Nausea and vomiting	1
Nerve palsy (transient)	5
Renal dysfunction (transient)	1
Significant swelling and/or pain	7
Skin injuries^a^	5
Subtotal	19
Total	27

^a^Includes blistering, drainage, and ulceration. ROM = range of motion

**Fig. 3:**
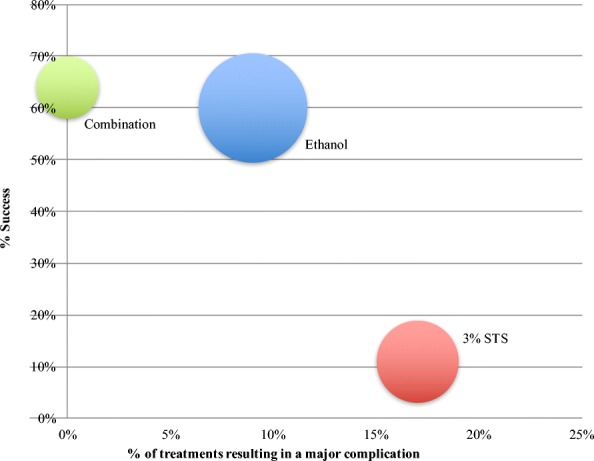
Success rate versus complication rate for each sclerosant. Size of dot corresponds to the number of times agent was used. Single treatment with bleomycin excluded

Overall, combination gave the highest success rate (64%) with the lowest major complication rate (0%) followed by ethanol with a 60% success rate and a 9% complication rate, while 3% STS had the lowest success rate (11%) and highest complication rate (17%), Fig. 3.

The total sclerosant volume per volume of VM was calculated for 17 patients with available images. The average VM volume was 156cm^3^ (range 3.0–1703.2cm^3^) with an average sclerosant amount of 42%*v*/v (range 5.4–99.1%*v*/v). There was no relationship between the amount of sclerosant and a success or failed treatment outcome, *p* = 0.281.

## Discussion

Our results indicate that combination, ethanol with 3% STS, had the highest success rate and lowest complication rate. Ethanol had a similar success rate but had more major complications, while 3% STS had the lowest success rate and highest major complication rate. Interim recurrences were found for 16 patients and no trend was found with respect to the type of sclerosant used. The greatest proportion of venous malformations was found in the head and neck region and had the highest percentage of successful outcomes. Similarly, Type I and II lesions had the most successful outcomes compared to Type III and IV. No trend was found between outcome and the amount of sclerosant administered per VM volume. It is possible that better results may have been achieved for patients with planned serial treatment had scheduling been optimal (Odeyinde et al., 2013). It may be that more treatments seen in non-responders reflect the intent to pursue sclerotherapy over other options until it is deemed truly unhelpful.

In comparison with literature, the location of VMs in our study was mainly in the head and neck, which correlates with previous literature (Ernemann et al., 2010). Additionally, our results found that type I and II lesions had the highest success rates, which correlates with previous findings of higher success rates for these lesions due to minimal or more physiological interactions with normal venous circulation (Stuart et al., 2015; Legiehn & Heran, 2008). Our study showed no trend between outcome and the amount of sclerosant administered per VM volume which may question previous reports that apply a general protocol of standard sclerosant volume per superficially measured lesion diameter (Alakailly et al., 2015). While literature has reported that ethanol is associated with the lowest incidence of lesion recurrence, no statistically significant difference in recurrence between sclerosants was found in this study (Su et al., 2010; Odeyinde et al., 2013; Puig et al., 2003). With respect to outcomes and complications, some clinicians have favoured ethanol as the primary agent due to its effectiveness, while others favour STS, due to safety concerns (Ernemann et al., 2010; Su et al., 2010; Prasetyono & Kreshanti, 2010; Odeyinde et al., 2013; Lee et al., 2003). Our results indicate that not only was ethanol more effective than 3% STS, it was also associated with fewer complications, with rates similar to previous reports (Stuart et al., 2015) and in contrast to others that suggest ethanol causes a greater proportion of complications (Berenguer et al., 1999; Odeyinde et al., 2013). By selectively targeting portions of the malformation that have surrounding anatomy that are more tolerant of ethanol and targeting other portions of the malformation with 3% STS in more anatomically sensitive areas at the same treatment session, we can achieve a better treatment effect with a better side-effect profile.

More factors than the sclerosant only may impact outcomes and complications including proximity to high contact areas such as plantar or palmar surfaces or areas of esthetic concern, as well as symptom tolerance and clinician training. A conclusion from a recent systematic review in 2016 reported that despite a lack of consensus for the most effective sclerosant, the sclerosing agent should be based on the risk of complications (Stuart et al., 2015). This, in part, explains the increased use of bleomycin in sclerotherapy practice. Overall, the results of this study combined with what is known from literature indicate that the pattern and approach to treatment should be tailored to the patient and not only take into account the efficacy of the sclerosant but also the nature of the VM.

### Limitations

This study was designed retrospectively and may have inherent information bias. The study population was small and thus multivariate analysis was not done. Patients receiving serial/staged treatments frequently experienced treatments intervals longer than the planned 3–4 months due to resource limitations (Odeyinde et al., 2013).

## Conclusion

In conclusion, our study found that Type I and II VMs and those located on the head and neck region had the highest success rates. The most effective sclerosants were combination and ethanol with higher success and lower complication rates than 3% STS. The efficacy of a sclerosant, location and nature of the VM should all be considered in treatment plans. Moving forward, future work should aim at studying large populations to assess the multiple variables that may influence treatment outcomes and complications.

## Abbreviations

STS: Sodium tetradecyl sulfate
VM: Venous malformation

## Authors’ information

JA, MH, and DC are members of the vascular anomalies clinic at BC Children’s Hospital, which cares for pediatric and adult patients. JA and DC are plastic surgeons and MH is an interventional radiologist. The vascular anomalies clinic team is also composed of a specialist nurse, an otolaryngologist, medical genetics, pediatrics, dermatology, orthopedics, occupational therapy.
